# Combining modern tracking data and historical records improves understanding of the summer habitats of the Eastern Lesser White‐fronted Goose *Anser erythropus*

**DOI:** 10.1002/ece3.7310

**Published:** 2021-03-09

**Authors:** Haitao Tian, Diana Solovyeva, Gleb Danilov, Sergey Vartanyan, Li Wen, Jialin Lei, Cai Lu, Peter Bridgewater, Guangchun Lei, Qing Zeng

**Affiliations:** ^1^ Center for East Asian‐Australasian Flyway Studies Beijing Forestry University Beijing China; ^2^ Institute of Biological Problems of the North Far East Branch Russian Academy of Sciences Magadan Russia; ^3^ Peter the Great Museum of Anthropology and Ethnography Russian Academy of Sciences St.‐Petersburg Russia; ^4^ North‐East Interdisciplinary Scientific Research Institute n. a. N. A. Shilo Far East Branch Russian Academy of Sciences Magadan Russia; ^5^ Department of Planning, Industry and Environment Environment Energy and Science Sydney NSW Australia; ^6^ Institute for Applied Ecology University of Canberra Canberra ACT Australia; ^7^ Advanced Wellness Research Centre Sheffield Hallam University Sheffield UK

**Keywords:** Arctic, eastern population, GPS tracking, Lesser White‐fronted Goose *Ansererythropus*, species distribution modeling, summer range

## Abstract

The Lesser White‐fronted Goose (*Anser erythropus),* smallest of the “gray” geese, is listed as Vulnerable on the IUCN Red List and protected in all range states. There are three populations, with the least studied being the Eastern population, shared between Russia and China. The extreme remoteness of breeding enclaves makes them largely inaccessible to researchers. As a substitute for visitation, remotely tracking birds from wintering grounds allows exploration of their summer range. Over a period of three years, and using highly accurate GPS tracking devices, eleven individuals of *A. erythropus* were tracked from the key wintering site of China, to summering, and staging sites in northeastern Russia. Data obtained from that tracking, bolstered by ground survey and literature records, were used to model the summer distribution of *A. erythropus*. Although earlier literature describes a patchy summer range, the model suggests a contiguous summer habitat range is possible, although observations to date cannot confirm *A. erythropus* is present throughout the modeled range. The most suitable habitats are located along the coasts of the Laptev Sea, primarily the Lena Delta, in the Yana‐Kolyma Lowland, and smaller lowlands of Chukotka with narrow riparian extensions upstream along major rivers such as the Lena, Indigirka, and Kolyma. The probability of *A. erythropus* presence is related to areas with altitude less than 500 m with abundant wetlands, especially riparian habitat, and a climate with precipitation of the warmest quarter around 55 mm and mean temperature around 14°C during June‐August. Human disturbance also affects site suitability, with a gradual decrease in species presence starting around 160 km from human settlements. Remote tracking of animal species can bridge the knowledge gap required for robust estimation of species distribution patterns in remote areas. Better knowledge of species' distribution is important in understanding the large‐scale ecological consequences of rapid global change and establishing conservation management strategies.

## Graphical abstract

The extreme remoteness of breeding enclaves makes the population largely inaccessible to researchers. In the study, we modeled summering range of the East Asian Lesser White‐front Goose *Anser erythropus* sub‐population by combining GPS tracking data, historical ground survey records, and literature sources, and confirmed a contiguous summer range, rather than patchy habitats suggested by earlier literatures.

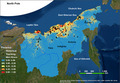

## 1 INTRODUCTION

The Lesser White‐fronted Goose *Anser erythropus* is the smallest of the so‐called “gray” geese of the genus *Anser* (BirdLife International, 2018). Excluding threatened taxa, gray geese are traditionally used for subsistence and sport hunting in Eurasia. Arctic nations especially continue to consider geese as a sustainable source of fresh meat in spring. However, hunting bans in many European countries, Republic of Korea and Japan have allowed the various species of gray geese to become part of agricultural landscapes. In contrast, several species of gray geese in China prefer to winter on wetlands with typically low levels of human use, rather than exploiting agricultural lands that are densely populated by people and their livestock (Deng et al., 2019). Following continuing population decline for decades, *A. erythropus* has been listed as globally Vulnerable in the IUCN Red List since 1994 (BirdLife International, 2018).

Three populations can be distinguished: Fennoscandian (Norway—Kola peninsula), the main western (NW Russia E of the White Sea—Taimyr Peninsula), and the eastern (E of Taimyr—Chukotka) with potential overlap zone of the breeding grounds between the main populations (Jones et al., 2008). Aarvak and Øien (2018) noted that the Fennoscandian population appeared on the brink of extinction with only 30–35 pairs left, despite active conservation efforts since early 1980s. After 2015 the Fennoscandian population has, however, somewhat increased to 40–50 pairs thanks to good reproduction years 2015–2016 (Marolla et al., 2019). In addition to these, there is a reintroduced small population in Sweden (Andersson & Holmqvist, 2010; Ruokonen et al., 2000). The number of the West Asian subpopulation assessed from counts at stop‐over sites during autumn migration has risen from an estimated 10,000–21,000 in early 2000s (Fox et al., 2010) to 30,000–34,000 in 2015 (Cuthbert et al., 2018) and perhaps as high as 48,580 ± 2,820 in 2017 (Rozenfeld et al., 2019). However, this increase could be attributed to additional survey efforts for *A. erythropus* at previously infrequently or unvisited staging sites in Kazakhstan. The most recent estimate of the East Asian subpopulation is 14,000–19,000 individuals (Jia et al., 2016), accounting for around 25% of the global *A. erythropus* population (Jia et al., 2016; Rozenfeld et al., 2019). The eastern subpopulation of *A. erythropus* extends from the Taymyr Peninsula eastward to Chukotka region (Cao et al., 2018; Lei, Jia, Zuo, et al., 2019; Morozov, 1995; Morozov & Syroechkovski‐Jr, 2002), and is declining (BirdLife International, 2018). A range of threats, including habitat loss and degradation along the migration route and on the wintering grounds proposed to fragmentation of the formerly continuous breeding range, have all been identified being responsible for past population declines (Grishanov, 2006; Madsen et al., 1984). In addition, illegal and accidental hunting (i.e., the genuine confusion with the similar looking Greater White‐fronted Goose *A. albifrons*, a species that can be hunted legally in Russia) are also threats to population viability.

**FIGURE 1:**
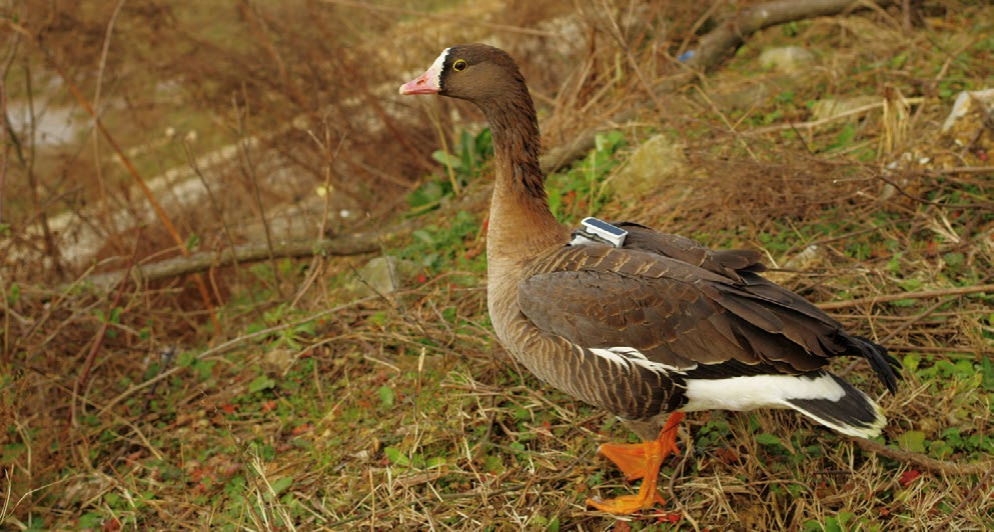
Lesser White‐fronted Goose *Anser erythropus* with a backpack GPS transmitter

**FIGURE 2:**
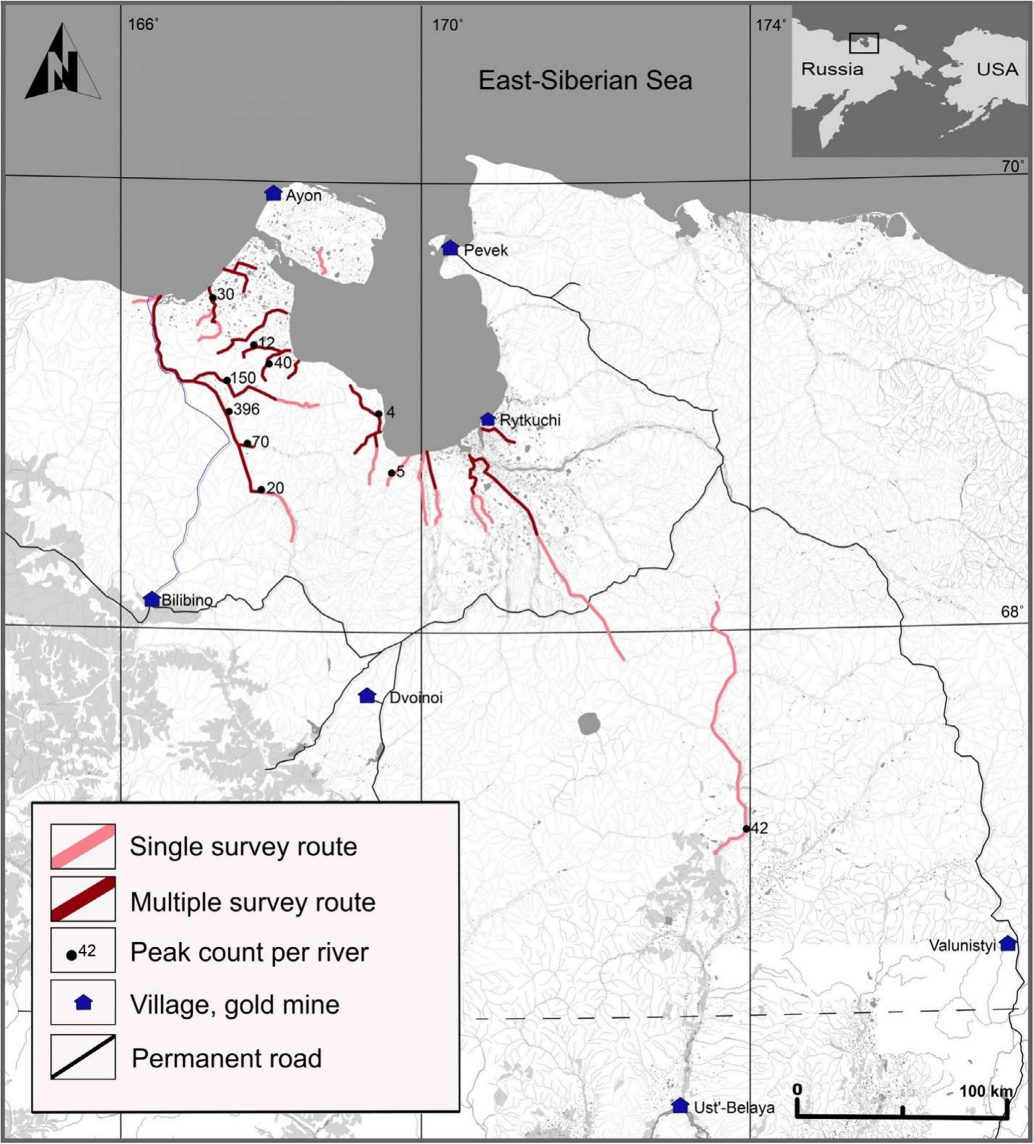
Survey route and peak counts of the Lesser White‐fronted Geese on the rivers of West Chukotka, 2002–2019

Quantitative knowledge of a species spatial distribution is the cornerstone for its effective conservation (Malahlela et al., 2019; Smeraldo et al., 2020). Due to the remoteness and restricted accessibility, historical observations of the summer range of the East Asian subpopulation are rather scarce (Lei, Jia, Zuo, et al., 2019; Malahlela et al., 2019; Morozov, 1995; Morozov & Syroechkovski‐Jr, 2002; Ruokonen et al., 2004; Smeraldo et al., 2020). Further, there are no systematic surveys covering the potential range of eastern subpopulation of *A. erythropus* (Supplementary S1). Current knowledge on the breeding distribution and habitat preference of *A. erythropus* is therefore limited (Egorov & Okhlopkov, 2007; Solovieva & Vartanyan, 2011). In the last 25 years, ornithologists generally considered that the East Asian *A. erythropus* had a patchy breeding distribution, and the number, position, and shape of those areas changed as new knowledge was acquired from occasional visits to remote sites in East Siberia as illustrated in Figure 1. Furthermore, an intensive multiyear survey in the area adjacent to the breeding grounds along the Rauchua River, West Chukotka, helped locate a number of breeding/molting groups and separated broods, suggesting that the entire survey area was populated by *A. erythropus* (Figure 2). This suggests that a single survey in one year, the usual method employed to study distribution of geese in remote areas of East Siberia (Egorov & Okhlopkov, 2007; Solovieva & Vartanyan, 2011), may not allow for an effective understanding of the summering distribution, limiting potential conservation actions for the species.

As new tracking technologies have developed, the investigation and quantification of spatial and temporal distributions of wide‐ranged migratory species, such as *A. erythropus*, now typically involve the deployment of telemetric tracking devices (Jiguet et al., 2011; Pimm et al., 2015). Rapid accumulation of tracking data offers new insights to assess distribution ranges and to explore habitat preferences (Kays et al., 2015). For example, tracking data can be linked with environmental conditions and used in ecological niche models to predict the overall space use by a population (Jiguet et al., 2011). In this context, this paper aimed to quantify to the potential summering range of the East Asian *A. erythropus* subpopulation by combining GPS tracking data, historical ground survey records, and literature sources. Using bioclimatic, geomorphological, land cover, and human disturbance layers, we used Maxent (a niche modeling technique, Phillips, 2006), to predict the summering habitats of *A. erythropus* within East Siberia in an ensemble forecast framework, that is, averaging predictions from many models (100 in this study) to account for data uncertainties and model variability (Pearson et al., 2006). Niche models using both historical records and recent tracking data could help to get better understanding of the summering distribution of the East Asian *A. erythropus* subpopulation and provide more accurate information for conservation plans including identifying potential threats and prioritizing management actions.

## 2 MATERIALS AND METHODS

### 2.1 Study area

The study area was in northeast Siberia, extending eastwards from Olenyok R (119.2 E) to the watershed between the Pacific and Arctic drainage basins, including Republic of Sakha, Magadanskaya Oblast, and Chukotskiy Autonomous Okrug. *A. erythropus* was never reported in the Arctic Archipelagos, these island areas are excluded in our study.

### 2.2 Surveys in West Chukotka, Russia

During July‐August 2002–2019 surveys were undertaken along rivers and lake habitats in the area of 19,260 km^2^ of assumed *A. erythropus* range in Chukotka (Figure 2). Brood‐rearing adult *A. erythropus* with their brood or flocks of molting adult *A. erythropus* were counted during downstream travel in a motorboat from the upper reaches of rivers, which were reached by helicopter. A description of the study area and survey results of 2002–2010 have been previously published (Solovieva & Vartanyan, 2011). No *A. erythropus* were found on lakes and only surveys along rivers have been used in this study (Figure 2). Positions and numbers of *A. erythropus* were given as (a) middle point and peak number for each river from surveys in multiply years; (b) middle point and number per river from single survey for the rivers surveyed once. As rivers of the study area are relatively short (up to 320 km) and uniform by habitat type, we considered each river as one data point for the niche modeling. These surveys provided 11 records for the model comprising eight breeding records and three molting records.

### 2.3 Data extraction from published sources

A total of 13 records of breeding or molting *A. erythropus* were compiled from historical surveys along the rivers dated after 1998. Originally 11 of these records were not attributed to GPS coordinates and to georeference them, we converted descriptions of records (river name and distance to the nearest village) to coordinates.

### 2.4 Capture methods and data tracking

Using techniques described in Lei, Jia, Zuo, et al. (2019), individual *A. erythropus* captured, during the winter of 2016/17 at East Dongting Lake, China. This lake is the most important wintering site for the species, supporting more than 70% of the East Asian subpopulation (Wang et al., 2012). A Total of 88 *A. erythropus* were captured and tagged by experienced hunters using baited clap traps, and 11 individuals returned with a completed wintering‐migration‐summering‐migration‐wintering cycle (Table 1). The tracking data for the rest 77 birds were not recovered either due to device malfunction or casualty.

**TABLE 1:** Summary of eleven tagged Lesser White‐fronted Geese used for this study

ID	Capture date	GPS start date	GPS end date	Nb days	Nb summers	Nb of GPS fixes
BFUL041	20.11.2016	23.11.2016	16.04.2018	509	1	7,227
BFUL044	30.11.2016	02.12.2016	09.06.2018	554	1	8,459
BFUL050	25.11.2016	27.11.2016	19.05.2018	538	1	8,351
BFUL057	30.11.2016	02.12.2016	17.07.2018	592	1	4,093
BFUL059	30.11.2016	02.12.2016	29.12.2017	392	1	4,050
BFUL065	05.12.2016	07.12.2016	05.09.2017	272	1	4,832
BFUL068	15.12.2016	16.12.2016	28.05.2018	528	1	9,347
BFUL051	25.11.2016	28.11.2016	25.12.2018	757	2	7,812
BFUL061	30.11.2016	02.12.2016	12.05.2019	891	2	11,490
BFUL074	15.01.2017	19.01.2017	14.05.2019	845	2	6,932
BFUL062	08.12.2016	11.12.2016	27.11.2019	1,081	3	17,848

Birds were fitted with transmitters (Hunan Global Messenger Technology Company, China) programmed to record GPS position and speed every 1–3 hr depending on the battery condition. Transmitters were solar powered to enable the global system for mobile communication (GSM) to transmit data *via* the short message service (SMS). These backpack design transmitters were 55 × 36 × 26 mm in size and weighed 22 g (appr. 1.6% of the bird's body mass; Lei, Jia, Zuo, et al., 2019). As Mobile network coverage is sparse or nonexistent in summering sites of northeast Russia, the stored data obtained from that area were downloaded when birds returned to China.

GPS records of locations (accuracy of <1,000 m) were used in the analysis of *A. erythropus* journeys to Russia. For nonbreeding *A. erythropus* (the longest one‐way migration recorded was 16,172 km in 60 days, Lei, Jia, Zuo, et al., 2019), it was assumed the spring migration turned to summering activities when the trans‐latitudinal movement became mostly trans‐longitudinal. Like spring migration, we assumed summering was terminated when a pronounced southbound movement was detected. For breeding birds, the date of arrival at a breeding site was used to indicate the start of summering. The site was classified as staging if the bird stayed at a location for more than four days.

### 2.5 Environmental predictors

To model the potential summering habitat, a range of environmental variables were used including bioclimatic, geomorphological, land production, and human disturbance.

#### 2.5.1 Bioclimatic

Bioclimatic variables were taken from the 30 s WorldClim (v2.1) climate data, downloaded from http://www.worldclim.org, which were generated through interpolation of monthly mean temperature and rainfall data from weather stations for the period of 1970–2000 (Fick & Hijmans, 2017; Hijmans et al., 2005). We selected five variables that are relevant to geese summering including Max Temperature of the Warmest Month (i.e., July, Bio5), Mean Temperature of Wettest Quarter (i.e., June‐August, Bio8), Mean Temperature of Warmest Quarter (i.e., June‐August, Bio10), Precipitation of Wettest Month (i.e., July, Bio13) and Precipitation of Warmest Quarter (i.e., June‐August, Bio18).

#### 2.5.2 Geomorphological

Topographic heterogeneity is important for species distribution (Austin & Van Niel, 2011). Three topographic variables were included in the modeling, namely elevation (digital elevation model, DEM), LDFG (local deviation from global mean) and TRI (terrain ruggedness index). The global 1 km resolution digital elevation model (DEM) for the study area was downloaded from (http://srtm.csi.cgiar.org/) and cropped with the study. Based on the DEM, LDFG and TRI were calculated as:

(1) $$\mathrm{LDFG}=y_{i}‐\bar{y}\;\;\;\;\;$$

where $\overset{‐}{y}$ is mean evaluation of the 3 by 3 window, and $y_{i}$ is the elevation of the focus grid. Positive LDFG values represent locations that are higher than the average of their surroundings, as defined by the neighborhood (ridges). Negative LDFG values represent locations that are lower than their surroundings (valleys). LDFG values near zero are either flat areas (where the slope is near zero) or areas of constant slope (where the slope of the point is significantly greater than zero).

(2) $$\mathrm{TRI}={\left(\sum {\left(Z_{c}‐Z_{i}\right)}^{2}\right)}^{1/2}\;\;\;\;\;\;\;\;$$

where *Z_c_* is the elevation of the central grid and *Z_i_* is the elevation of one of the eight neighboring grids. The terrain ruggedness index (TRI) is a topographic measurement developed by Riley et al. (1999) to quantify topographic irregularities in a region.

As *A. erythropus* is ecologically dependent on wetlands and often observed breeding along river valleys (Solovieva & Vartanyan, 2011), we included a layer of distance to streams in the modeling. We generated the raster using polylines in the Global River Widths from Landsat (GRWL) dataset (Allen & Pavelsky, 2018) as the central lines. The polylines were checked to be a good represent of the rivers in the study area.

#### 2.5.3 Land production

To characterize land production, we calculated three variables (EVI_max_, EVI_hom,_ and EVI_range_) using EVI (Enhanced Vegetation Index) time series (2000–2009). The 10‐day global EVI images with 333 × 333 m resolution were downloaded from Copernicus Global Land Service (https://land.copernicus.eu/global/products/ndvi, data downloaded on 28 August 2019). EVI_max_ is an indicator of peak land productivity and was calculated as the 10‐year mean of annual max EVI. EVI_range_ is the range of land productivity (i.e., EVI_max_ − EVI_min_). EVI_hom_ is the similarity of EVI between adjacent eight pixels, and was computed as (Tuanmu & Jetz, 2015):

(3) $${\mathrm{EVI}}_{\mathrm{hom}}=\sum_{i,j=1}^{m}\frac{P_{i,\;j}}{1+{\left(i‐j\right)}^{2}}$$

where *m* is the number of all possible scaled EVI values (i.e., 100) and $P_{i,\;j}$ is the probability that two adjacent pixels have scaled EVI values of *i* and *j*, respectively. Both EVI_hom_ and EVI_range_ can be indicator of habitat diversity.

#### 2.5.4 Human disturbance

Human disturbance can lead to declines and local extinctions of avian species as well as habitat loss (Vollstädt et al., 2017). The inclusion of human disturbance data can increase the performance and accuracy of SDM (species distribution model ‐ Stevens & Conway, 2020). We compiled a database of all human settlements including villages and towns in the study area (i.e., Republic of Sakha, Magadanskaya Oblast, and Chukotskiy Autonomous Okrug) and generated a layer of distance to settlements as a proxy of human disturbance. Settlements with zero registered inhabitants (abandoned and closed before 2011) were excluded.

#### 2.5.5 Land cover

Forcey et al. (2011) found that land use has strong effects on waterbird distribution, and the percentage of waterbird abundance is positively related to the area of wetland. In this study, we used the 2015 global land cover map derived from satellite observations by Land Cover Climate Change Initiative (CCI) and available from https://maps.elie.ucl.ac.be/CCI/viewer/download.php. The map classifies the global terrestrial system into 28 major classes using United Nations Food and Agriculture Organization's land cover classification system (Di Gregorio, 2005).

R (R Core Team, 2019) packages “raster” (Hijmans et al., 2015) and spatialEco (Evans & Ram, 2018) were used for raster manipulation and calculation.

### 2.6 Modeling

A total of 96 georeferenced records were compiled by combining the tracking data and historical surveys (post‐1999) (Supplementary S2). To analyze the potential breeding range, maximum entropy implemented in the Maxent package (version 3.4.1) was used. Maxent is among the most robust and accurate SDM techniques (Elith et al., 2006, 2011; Kaky et al., 2020; Raffini et al., 2020). In the past two decades, it has gained popularity in conservation studies, partly because the technique is less sensitive to the number of recorded sites and uses presence‐only data (Elith et al., 2011). In developing the SDM, the program was set to take 75% of the occurrence records randomly for model training and the remaining 25% for model testing. The mean area under the receiver operating characteristic curve (AUC) was used to evaluate model performance, and AUC values >0.75 are considered as suitable for conservation planning (Lobo et al., 2008). The modeling process was replicated 100 times and we reported the mean as summering ranges to reduce the sampling bias (Merow et al., 2013).

Although collinearity is less of a problem for machine learning methods in comparison with statistical methods (Elith et al., 2011), minimizing correlation among predictors prior to model building is recommended (Merow et al., 2013). We used VIF (Variance inflation factor) to select predictors (Dupuis & Victoria‐Feser, 2013). Nine variables with VIF less than 10, including two bioclimatic variables (Bio10 and Bio18), two topographic variables (DEM and LDFG), two productivity variables (EVI_hom_ and EVI_range_), land cover, Distance to stream, and Distance to settlement, were included in model building.

Using the logistic outputs of MaxEnt, we applied the minimum training presence threshold (MTP) to produce binary habitat map. MTP threshold finds the lowest predicted suitability value for an occurrence point and ensures that all occurrence points fall within the area of the resulting binary model (Elith et al., 2011).

## 3 RESULTS

### 3.1 Potential summering range of the East Asian subpopulation of *A. erythropus*

The mean training AUC of the 100 models was 0.9510 suggested these models are very useful (Swets, 1988) for predicting the summering range of *A. erythropus*. The standard deviation of AUC was very small (0.0007) indicating the models were stable. Moreover, the mean testing AUC was 0.9356 (*SD* = 0.0739), which was comparable to the training AUC, suggesting excellent predictive power of the fitted model.

**FIGURE 3:**
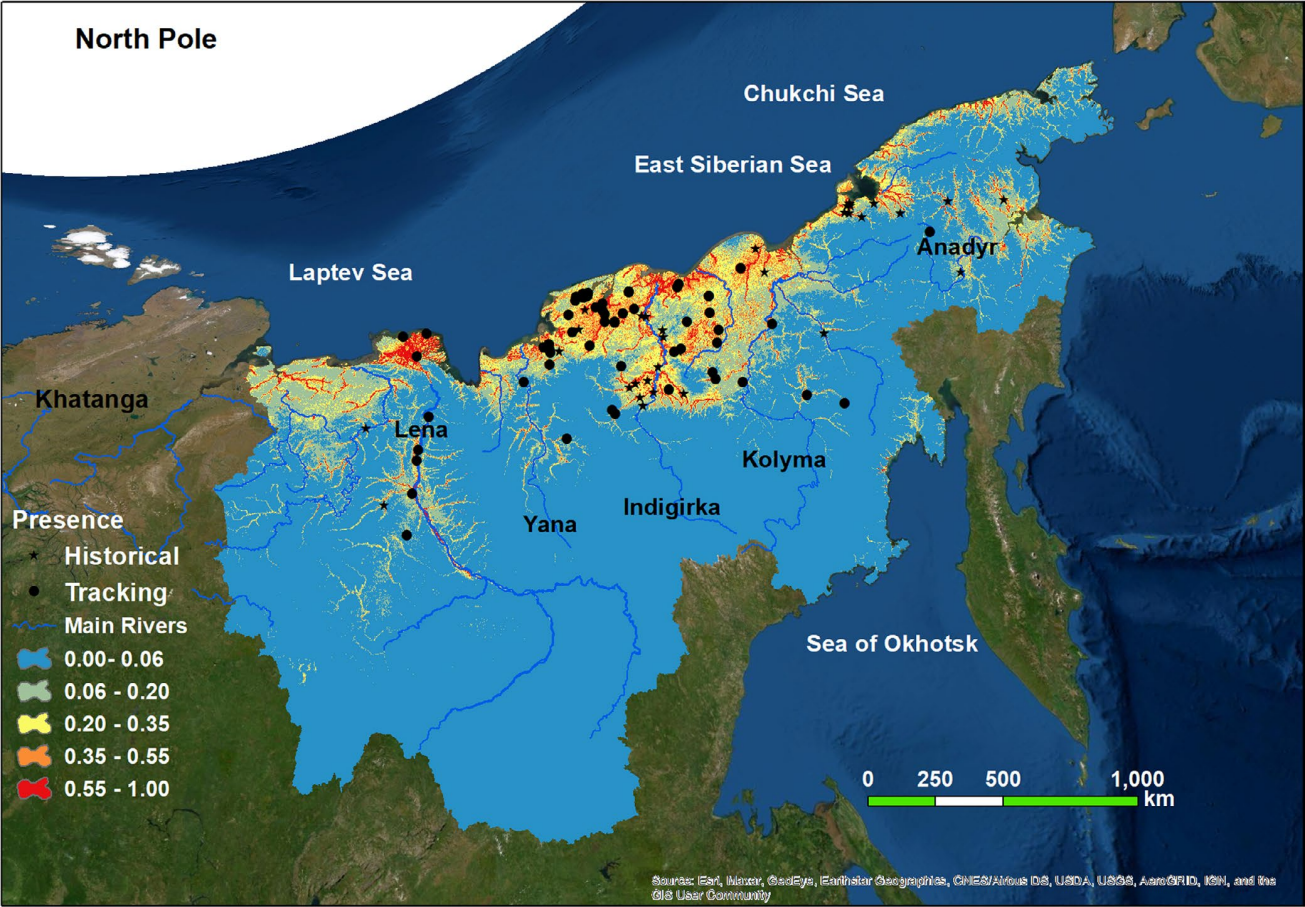
Fitted Maxent model showing the probability of summering habitats of the Eastern population of the Lesser White‐fronted Goose. Red color indicates the strongest probability, with orange and yellow less so. Background: Aerial Imagery from ESRI (http://services.arcgisonline.com/arcgis/rest/services). Projection: Asia North Albers Equal Area Conic

**FIGURE 4:**
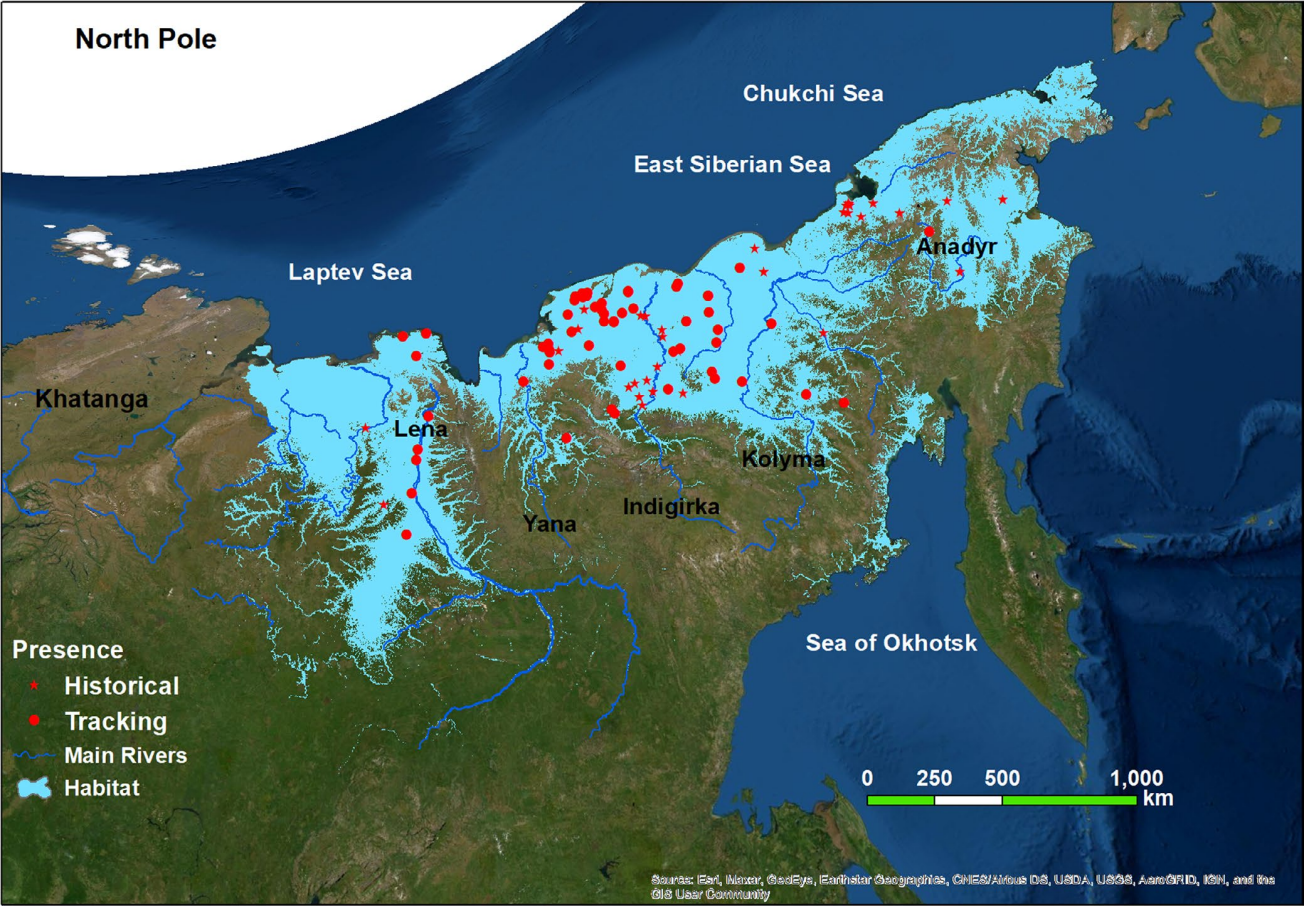
Breeding and molting habitats of the Eastern population of the Lesser White‐fronted Goose based on the minimum training presence threshold. Projection: Asia North Albers Equal Area Conic. Background: World Imagery from ESRI (http://services.arcgisonline.com/arcgis/rest/services)

The average of summering distribution prediction of the 100 models was presented in Figure 3. The most suitable habitats are located along the coasts of the Laptev Sea, primarily the Lena Delta, in the Yana‐Kolyma Lowland, and smaller lowlands of Chukotka with narrow strips extended upstream to catchments of major rivers such as the Lena, Indigirka, and Kolyma (Figure 3). The binary map (Figure 4) produced using the criteria of minimum training presence threshold indicated that 36.44% of the study area was suitable summering habitats.

Lowland wetlands including large deltas, estuaries, tundra, and swampy floodplains (i.e., floodplain containing numerous ponds and shallow lakes), which extend from the Lena Delta at the west to the Kolyma River at the east, provide the most extensive and continuous breeding and molting ground for *A. erythropus* in our study area (Figures 3 and 4). This is particularly the case for the very large Lena Delta, (~29,000 km^2^) where the predicted summering habitats include tundra together with numerous interlaced channels and lakes.

Most of predicted breeding habitats are covered by a range of plant types including grasses, sedges, herbs, as well as abundant mosses and lichens. This tundra vegetation is also characterized by widely spaced shrubs (e.g., *Betula nana (s.l.), Dushecia fruticosa*, and several species of *Salix*). Such tundra vegetation along major rivers within the taiga biome also have potential to be suitable habitat (Figure 3).

### 3.2 Effects of environmental factor on the summering range of *A. erythropus*

Of the nine environmental variables included in model building, elevation was the most important, strongly contributing to the scaling of the Maxent model (59.4% based on the model gain and 54.3% based on re‐evaluation of the random permutation of training presence and background data, Table 2). Other highly influential variables (with more than 5% permutation contribution) include precipitation of the warmest quarter, distance to streams, and mean temperature of the warmest quarter (Table 2).

**TABLE 2:** Relative contributions of the environmental variables to the breeding habitat distribution of *A. erythropus* ranked by permutation importance

Predictor	Percent contribution	Permutation importance
Elevation	59.4	54.3
Precipitation of warmest quarter	5.0	25.2
Distance to streams	20.3	6.5
Mean temperature of warmest quarter	5.2	5.8
Range_EVI	0.9	2.6
Distance to settlement	2.4	2.2
Land cover	5.5	2.0
Homogeneity_EVI	1.0	0.8
Local deviation from global	0.2	0.6

Although highly correlated environmental predictors were excluded from model fitting, there are still collinearities in the remaining variables. For example, the Pearson r between Bio10 (precipitation of the warmest quarter) and Bio18 (mean temperature of the warmest quarter) is relatively high (−0.82) in the study area. Thus, the variable contributions in Table 2 should be interpreted with caution.

**FIGURE 5:**
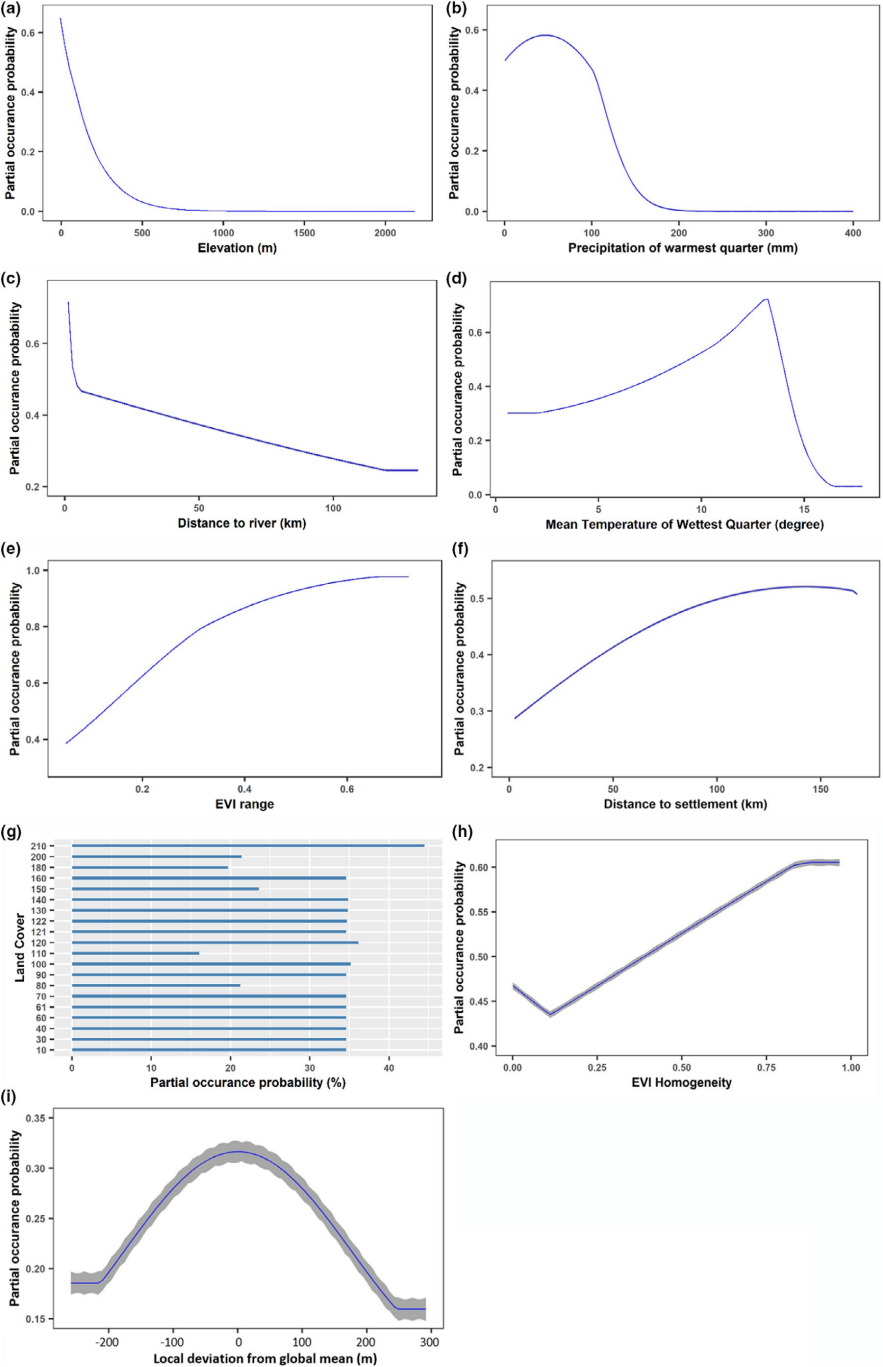
The relationships between the probability of *A. erythropus* occurrence and the top ten environmental variables based on permutation. Blue lines are mean response curves, and gray shades are 1 standard deviation

The marginal effects of the predictors on habitat suitability of *A. erythropus* (i.e., occurrence probability responds to changes in a specific explanatory variable while other covariates are assumed to be held constant as mean) were presented in Figure 5. The response curves showed that the effects of environmental factors on the occurrence of *A. erythropus* were strongly nonlinear.

For topographic variables, the probability of *A. erythropus* presence declines with increasing elevation up to 500 m, with locations higher than 500 m elevation were virtually devoid of *A. erythropus* (Figure 5a). Also, the response curve of LDFG indicated that the geese prefer relatively flat sites (Figure 5i). In terms of bioclimatic variables, the probability of *A. erythropus* presence increases with precipitation of the warmest quarter to around 55 mm and mean temperature of the warmest quarter to around 14°C, after which there is a sharp decrease (Figure 5b,d). Human disturbance also influences summering habitat, with suitability increasing the further the site is from human settlement (Figure 5f). The response curve of habitat occurrence probability to distance from rivers (Figure 5c) suggests that the geese were highly dependent on wetlands and riparian areas (Figure 5c). Within the riparian zone, the summering habitat suitability decreases sharply with increasing distance from water courses, and after about 4.5 km virtually no birds are found. *A. erythropus* generally prefers land cover types waters (code 210) and shrubland (120; Figure 5g). The modeling results suggest that the probability of occurrence increases with land productivity range (Figure 5e) and homogeneity (Figure 5h).

## 4 DISCUSSION

Due to the remoteness and restricted accessibility, there are few historical observations of the summering ground of this population (Ruokonen et al., 2004), and our current knowledge on the breeding distribution and habitat preference is limited (Supplementary S1 and see Artiukhov & Syroechkovski‐Jr, 1999; Egorov & Okhlopkov, 2007; Solovieva & Vartanyan, 2011). In this context, rapid development of animal tracking technologies offers new insights to determine distribution range and habitat preferences (Kays et al., 2015). In this study, we combined historical records with recent tracking data to model potentially suitable areas of the east subpopulation of *A. erythropus* across the more than 7,400,000 km^2^ of arctic and subarctic of northeastern Russia.

Our findings assist conservation of this threatened species by identifying the most suitable breeding grounds and assessing existing and future threats. As *A. erythropus* often co‐occurs with other geese (e.g., Greater White‐fronted Goose (*A. albifrons*), Bean Goose (*A. fabalis*), and Brent Goose (*Branta bernicla*) and other waterfowl including ducks and tundra swan (Hodges & Eldridge, 2001; Krechmar & Kondratiev, 2006; Pozdnyakov, 2002), the breeding habitat map could also be used for prioritizing waterbird conservation including through identification of high‐priority conservation areas.

### 4.1 Model accuracy and breeding range

In recent years, animal tracking point data have been used in SDM construction either through direct use for model fitting (Williams et al., 2017) or for validating the output of the model (Pinto et al., 2016). By combining three‐year tracking data and historical surveys, our dataset represents the most comprehensive presence record and offers a solid basis to delineate the breeding range of the poorly known eastern subpopulation of *A. erythropus*. The cross‐validation results showed that the training and testing AUC are both high (i.e., greater than 0.92) and comparable, suggesting that the output is highly reliable (Phillips & Dudík, 2008).

The Maxent output suggested a continuous rather than patchy potential breeding and molting range of the *A. erythropus* on the plains adjusted to the Laptev, East Siberian, and Chukchi Seas and in the Anadyr Lowland. Within this over 4,000 km area of coastal plains, the Lena Delta, the wide Yana‐Kolyma Lowland, and smaller lowlands of Chukotka represent the most extensive breeding area with the highest probability of occurrence (Figures 3 and 4). While there are suggestions that breeding ranges of West and East Asian subpopulations overlap between 103 and 118 E, our work did not confirm this. The flat and rolling subarctic tundra is among the most productive wetland system in northeastern Russia (Gilg et al., 2000). Vegetation characteristic in this area is typical tundra, southern tundra with shrubs and forest‐tundra with sparse patches of larch (*Larix* spp.) Yurkovskaya (2011). A current IBA (Important Bird Area), including the four main deltas (i.e., the Kolyma, Indigirka, Yana, and Lena), covers about 34% of the modeled breeding range (BirdLife International, 2017). However, the majority of the coastal plains, extending up to 450 km inland (Figures 3 and 4), and valleys of large rivers are not included in this IBA. Although there are several Wetlands of International Importance under the Ramsar Convention on the Kamchatka Peninsula, the closest to the study area (Parapolsky Dol) does not contain habitat the modeling suggests as suitable. Highly suitable habitats in the study areas have legal protection through declaration as Federal (State) Nature Reserves: Ust‐Lenskiy, Olekminskiy and Magadanskiy, and also by Kytalyyk and Beringia National Parks.

### 4.2 Environmental characteristics of breeding habitat

The selection of environmental variables is a critical step in species distribution models (Araujo & Guisan, 2006; Fourcade et al., 2018), and hundreds of environmental factors have been utilized in Maxent (Bradie & Leung, 2017). These predictor variables can be loosely grouped into four main groups: limiting factors that control the ecophysiology of the species concerned (e.g., temperature, precipitation, pH); resource factors (e.g., vegetation, water areas), which are supplies needed by the organisms to survive; disturbance factors including anthropogenic and natural perturbations in the environment; and landscape factors, which can be related to the species dispersal limitations (Guisan & Thuiller, 2005; Vuilleumier & Metzger, 2006).

The geomorphological predictors (i.e., elevation, distance to streams and local deviation from global) collectively contributed to 61.4% of the model gain based on permutation test. This level of relative importance was considered very high for Maxent modeling (Bradie & Leung, 2017). The decisive role of topography in controlling the distribution of summering grounds might be attributed to strong preference of river valleys and lowlands, especially considering reduced mobility of geese during breeding and molting periods (Akesson & Raveling, 1982). Kosicki (2017) demonstrated the importance of topography for modeling the distribution of both lowland and upland bird species, and omitting topographic variables could lead to substantial overestimation of distribution range, especially for rare species. The response curves show that *A. erythropus* selects lowlands with a concave shape as preferred habitat, which is consistent with field observations (e.g., Artiukhov & Syroechkovski‐Jr, 1999; Egorov & Okhlopkov, 2007; Solovieva & Vartanyan, 2011), which reported the bird bred and molt in river valleys.

The majority of Maxent models include climate variables as limiting factors, and most studies found temperature and precipitation were very important variables (Bradie & Leung, 2017) as climate is believed to be the most important factor for species distributions (Gaston, 2003; Pasquale et al., 2020; Zhang et al., 2019). It is therefore not surprising that climate variables including precipitation and temperature were also important for *A. erythropus*. A significant finding of the study is that there was an optimal window of mean summer temperature in 9–14°C (Figure 5d) and dry continental or high Arctic precipitation of the wettest quarter in 55 mm (Figure 5b), within which the habitat suitability is maximized.

Land cover is also important and contributes strongly to model performance (Table 2). The response curve indicates that two land cover types are favored by *A. erythropus* including shrubland and open‐water areas. The land cover preference can be linked to the requirement of nest shelters during breeding season (Hilton et al., 2004) and food resources. In terms of food resources, the *A. erythropus* is an herbivorous browser, that is, it tends to increase the portion of the selective resources in their feeding range (Markkola et al., 2003). The wet sedge meadows on the alluvial floodplains that are preferred by herbivorous geese (Sedinger & Raveling, 1984), and are critical for brood rearing (Markkola et al., 2003) offer a range of highly nutritious species with an adequate protein–water ratio and low portions of cellulose and lignin, (e.g., grasses *Puccinellia phryganodes*, *Phragmites australis*, and sedges *Carex* spp.).

Finally, the most suitable habitats had higher land productivity heterogeneity (Figure 5e,h) which was expected as species richness and abundance often increases with habitat diversity (Chasko & Gates, 1982; Wen et al., 2015). Although human disturbance can sometimes increase diversity in such wetland systems, here the habitat suitability decreases with human disturbance (Figure 5f), reflecting the negative impacts of human presence (Lei, Jia, Wang, et al., 2019).

### 4.3 Conservation challenges

The results of this study highlight a major challenge from future climate change on the *A. erythropus*. First, many climate change models predict increasing spring temperatures and earlier snow melting (IPCC, 2014), which will lead to flooding, submergence, permafrost erosion, and loss and change in low‐lying coastal wetlands (Prowse et al., 2006). As the predicted summering habitats were concentrated in the lowland coastal zone of the Laptev and East Siberian Seas, the projected sea level rise (IPCC, 2014; Wrona et al., 2016) and increasing river flows (Karlsson et al., 2012; Wrona et al., 2016) could cause extensive habitat loss. The response curves of habitat suitability to topographic variables suggest that the relatively hilly and rugged landscape would restrict extension of suitable habitat landward and such “habitat squeeze” (Leo et al., 2019) would be highly detrimental to *A. erythropus*. Second, the models suggested that there was an “optimal window” in terms of mean summer temperature and precipitation, which could be interpreted as the realized climatic niche of *A. erythropus* (Merow et al., 2016). Rising temperatures under future climate change scenarios means that the temperature niche could shift northerly, which is sea. Third, studies have shown that encroachment of shrubs following projected climate change (e.g., *Salix ovalifolia* and *Dushecia fruticosa*) into the wet meadows (Carlson et al., 2018), would likely decrease quantity and quality of available food resources.

Finally, there is the threat from increasing anthropogenic disturbance; *A. erythropus* avoids locations near active mines (although can colonize such areas after mining is finished) (Egorov & Okhlopkov, 2007; Solovieva & Vartanyan, 2011). Currently, human population levels in the predicted summering range is among the lowest in the world, and the coastal areas of this region are some of the least explored. However, the coast of the Russian Arctic is likely to undergo rapid development as there are reserves of oil, gas, metals, and other natural resources which could be exported, with additional infrastructure, through the northeast Passage to European and Asian ports (Martini et al., 2019), more information on these potential developments can be found at http://ecoline‐eac.com/proekty/peschanka/deposit.html), and these developments present perhaps the most difficult challenges to the future of eastern subpopulation of *A. erythropus*.

## CONFLICT OF INTEREST

None declared.

## AUTHOR CONTRIBUTIONS

**Haitao Tian:** Formal analysis (equal); investigation (equal); writing‐original draft (lead). **Diana Solovyeva:** Conceptualization (equal); formal analysis (equal); investigation (equal); writing‐original draft (lead). **Gleb Danilov:** Formal analysis (equal); investigation (equal). **Sergey Vartanyan:** Formal analysis (equal); investigation (equal). **Li Wen:** Formal analysis (equal); writing‐original draft (equal); writing‐review & editing (equal). **Jialin Lei:** Formal analysis (equal); investigation (equal). **Cai Lu:** Formal analysis (equal); project administration (lead). **Peter Bridgewater:** Writing‐review & editing (equal). **Guangchun Lei:** Conceptualization (equal); funding acquisition (lead). **Qing Zeng:** Conceptualization (equal); formal analysis (equal); writing‐original draft (equal); writing‐review & editing (equal).

## ETHICAL APPROVAL

All field methods used in this study were approved by the Forestry Department of Hunan Province of China under scientific research license (No.11 Xiang Forest Protection (2014)). Field research was conducted with permission from the Bureau of East Dongting National Nature Reserve.

## Supporting information

Supplementary Material

Figure S1

## Data Availability

All data for analysis is available in Dryad (https://doi.org/10.5061/dryad.f7m0cfxvf).
