# Supplementary material for: Combining modern tracking data and historical records improves understanding of the summer habitats of the Eastern Lesser White‐fronted Goose Anser erythropus
Source: Ecol Evol. 2021 Mar 9;11(9):4126–39. doi: 10.1002/ece3.7310 (PMC8093674; doi:10.1002/ece3.7310)
Supplement: Supplementary file 1 — Supplementary Material [file ECE3-11-4126-s001.docx]

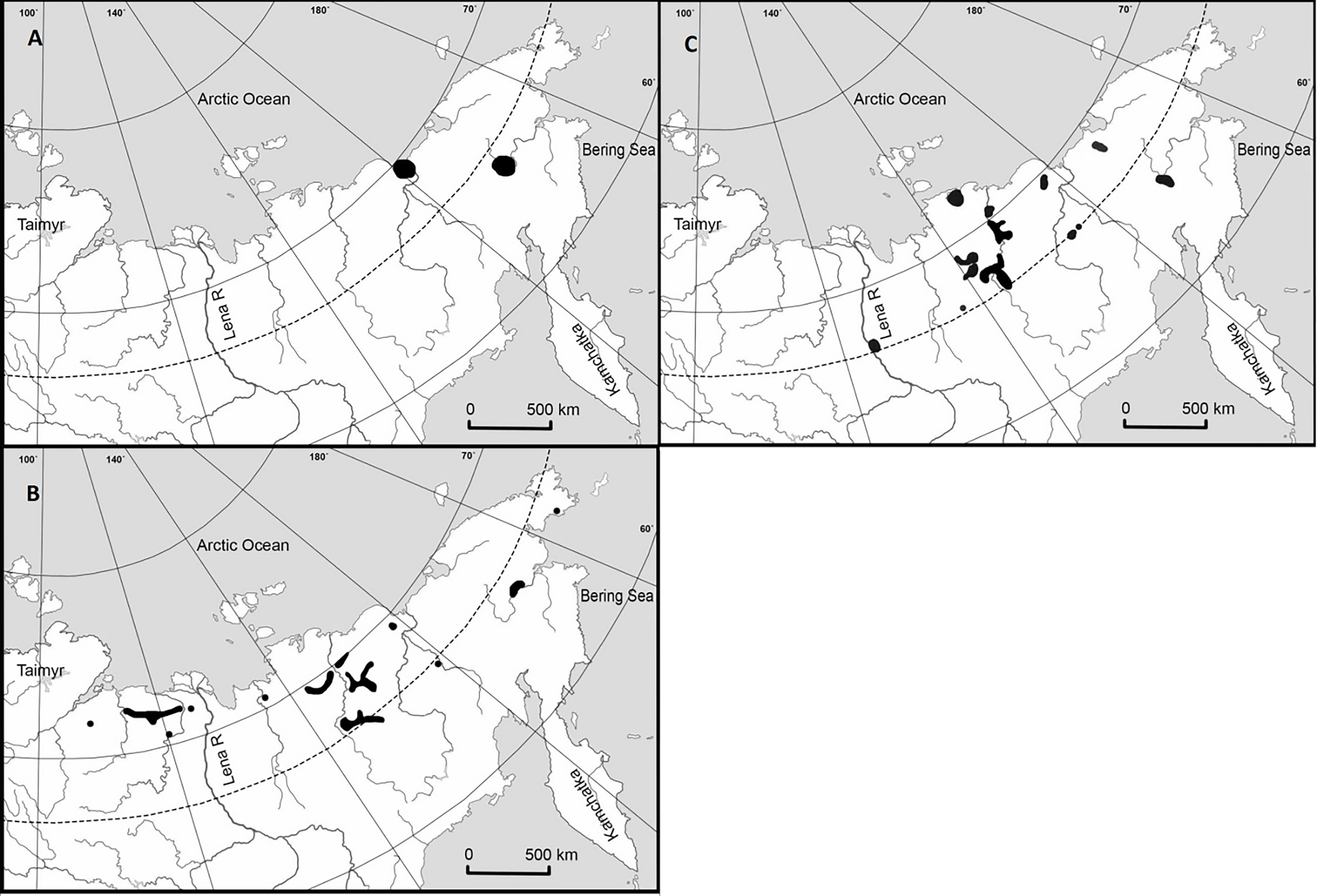


Supplementary 1 Historical summer (breeding and molting) range of the eastern sub-population of Lesser White-fronted Goose. Black contours indicate known breeding or molting enclaves. A) from Morozov 1995; B) from Morozov and Syroechkovski-Jr 2002; and C) from Cao *et al*. 2018.

Supplementary 2. Datasets for the model of summer range of East Asian Lesser White-fronted Goose

| Longitude | Latitude | Source | |
| --- | --- | --- | --- |
| 120.317942 | 67.73806 | Egorov and Okhlopkov 2007 | |
| 120.487306 | 70.547614 | Degtyaryev et al. 2013 | |
| 121.48454 | 66.53709 | our tracking | |
| 122.80895 | 67.85089 | our tracking | |
| 123.96738 | 68.92493 | our tracking | |
| 124.36163 | 69.25285 | our tracking | |
| 126.13753 | 70.29204 | our tracking | |
| 126.18952 | 73.48012 | our tracking | |
| 126.83646 | 72.58912 | our tracking | |
| 128.41763 | 73.30483 | our tracking | |
| 135.20029 | 70.24612 | our tracking | |
| 136.62152 | 67.7738 | our tracking | |
| 137.93828 | 70.44067 | our tracking | |
| 138.08054 | 71.104 | our tracking | |
| 138.42004 | 70.95199 | our tracking | |
| 138.43683 | 70.8315 | our tracking | |
| 138.48387 | 70.95029 | our tracking | |
| 138.59926 | 71.11974 | our tracking | |
| 138.63283 | 71.1142 | our tracking | |
| 139.238962 | 70.724368 | Bysykatova and Krapu 2009 | |
| 141.03751 | 71.14642 | our tracking | |
| 141.07106 | 67.99836 | our tracking | |
| 141.19348 | 67.84321 | our tracking | |
| 141.44843 | 71.78763 | our tracking | |
| 141.70633 | 71.12975 | Bysykatova and Krapu 2009 | |
| 141.88008 | 70.3949 | our tracking | |
| 142.62907 | 72.15652 | our tracking | |
| 142.8528 | 72.28665 | our tracking | |
| 143.082309 | 71.684090 | Bysykatova and Krapu 2009 | |
| 143.28883 | 68.4326 | Artiukhov and Syroechkovsky 1999 | |
| 143.42484 | 72.10284 | our tracking | |
| 143.50279 | 69.22856 | our tracking | |
| 143.55418 | 72.256 | our tracking | |
| 143.574 | 67.63742 | Artiukhov and Syroechkovsky 1999 | |
| 143.6705 | 67.9488 | Artiukhov and Syroechkovsky 1999 | |
| 143.67754 | 72.11861 | our tracking | |
| 143.68736 | 72.11787 | our tracking | |
| 143.80556 | 72.09962 | our tracking | |
| 143.80556 | 72.09962 | our tracking | |
| 143.86734 | 72.18413 | our tracking | |
| 143.87555 | 72.18348 | our tracking | |
| 143.8896 | 68.44833 | Artiukhov and Syroechkovsky 1999 | |
| 143.97899 | 71.55866 | our tracking | |
| 144.04825 | 72.17364 | our tracking | |
| 144.09372 | 70.93246 | our tracking | |
| 144.4334 | 71.33479 | our tracking | |
| 144.44122 | 71.16573 | our tracking | |
| 144.69682 | 71.56277 | our tracking | |
| 144.82095 | 70.74279 | our tracking | |
| 144.84357 | 70.74917 | our tracking | |
| 144.89071 | 67.88036 | Artiukhov and Syroechkovsky 1999 |  |
| 144.9222 | 68.3442 | Artiukhov and Syroechkovsky 1999 |  |
| 145.87618 | 70.88199 | our tracking |  |
| 146.09311 | 67.70303 | our tracking |  |
| 146.2928 | 68.5711 | Artiukhov and Syroechkovsky 1999 |  |
| 146.99821 | 70.82262 | our tracking |  |
| 147.0006 | 67.3311 | Artiukhov and Syroechkovsky 1999 |  |
| 147.26582 | 70.46797 | Artiukhov and Syroechkovsky 1999 |  |
| 147.35072 | 71.4397 | our tracking |  |
| 147.35565 | 71.46187 | our tracking |  |
| 147.5707 | 70.3344 | Artiukhov and Syroechkovsky 1999 |  |
| 147.9902 | 69.3829 | Artiukhov and Syroechkovsky 1999 |  |
| 148.09239 | 68.73299 | our tracking |  |
| 148.2271 | 69.6253 | Artiukhov and Syroechkovsky 1999 |  |
| 148.4669 | 68.6655 | Artiukhov and Syroechkovsky 1999 |  |
| 148.70873 | 68.70338 | our tracking | |
| 149.866 | 67.17923 | our tracking | |
| 149.97418 | 67.42832 | our tracking | |
| 150.40019 | 69.42544 | our tracking | |
| 151.34047 | 70.65373 | our tracking | |
| 151.59213 | 68.20238 | our tracking | |
| 151.59421 | 70.71585 | our tracking | |
| 151.62549 | 66.60401 | our tracking | |
| 152.32874 | 68.5559 | our tracking | |
| 152.50731 | 69.24902 | our tracking | |
| 153.27577 | 69.75746 | our tracking | |
| 153.27577 | 69.75746 | our tracking | |
| 155.2758 | 65.02602 | our tracking | |
| 156.33081 | 67.67036 | our tracking | |
| 156.94275 | 69.93875 | our tracking | |
| 157.27979 | 64.08596 | our tracking | |
| 158.43314 | 69.29271 | Andreev 2001 | |
| 159.09527 | 70.1631 | Andreev 2001 | |
| 159.35077 | 66.34719 | Andreev 2001 | |
| 167.12938 | 69.13715 | our survey data | |
| 167.404538 | 68.98925 | our survey data | |
| 167.691378 | 69.249261 | our survey data | |
| 167.904236 | 69.154175 | our survey data | |
| 167.943669 | 68.584661 | our survey data | |
| 167.95713 | 69.26019 | our survey data | |
| 167.96771 | 69.201373 | our survey data | |
| 169.51087 | 68.64722 | our survey data | |
| 170.55549 | 67.77368 | our survey data | |
| 170.74093 | 64.90449 | Andreev 2001 | |
| 171.22899 | 66.60868 | our tracking | |
| 174.038992 | 66.905752 | our survey data | |
| 177.23504 | 65.55287 | Heinicke *et al* 2009 | |
