# Supplementary figures and images for: Combining modern tracking data and historical records improves understanding of the summer habitats of the Eastern Lesser White‐fronted Goose Anser erythropus
Source: Ecol Evol. 2021 Mar 9;11(9):4126–39. doi: 10.1002/ece3.7310 (PMC8093674; doi:10.1002/ece3.7310)

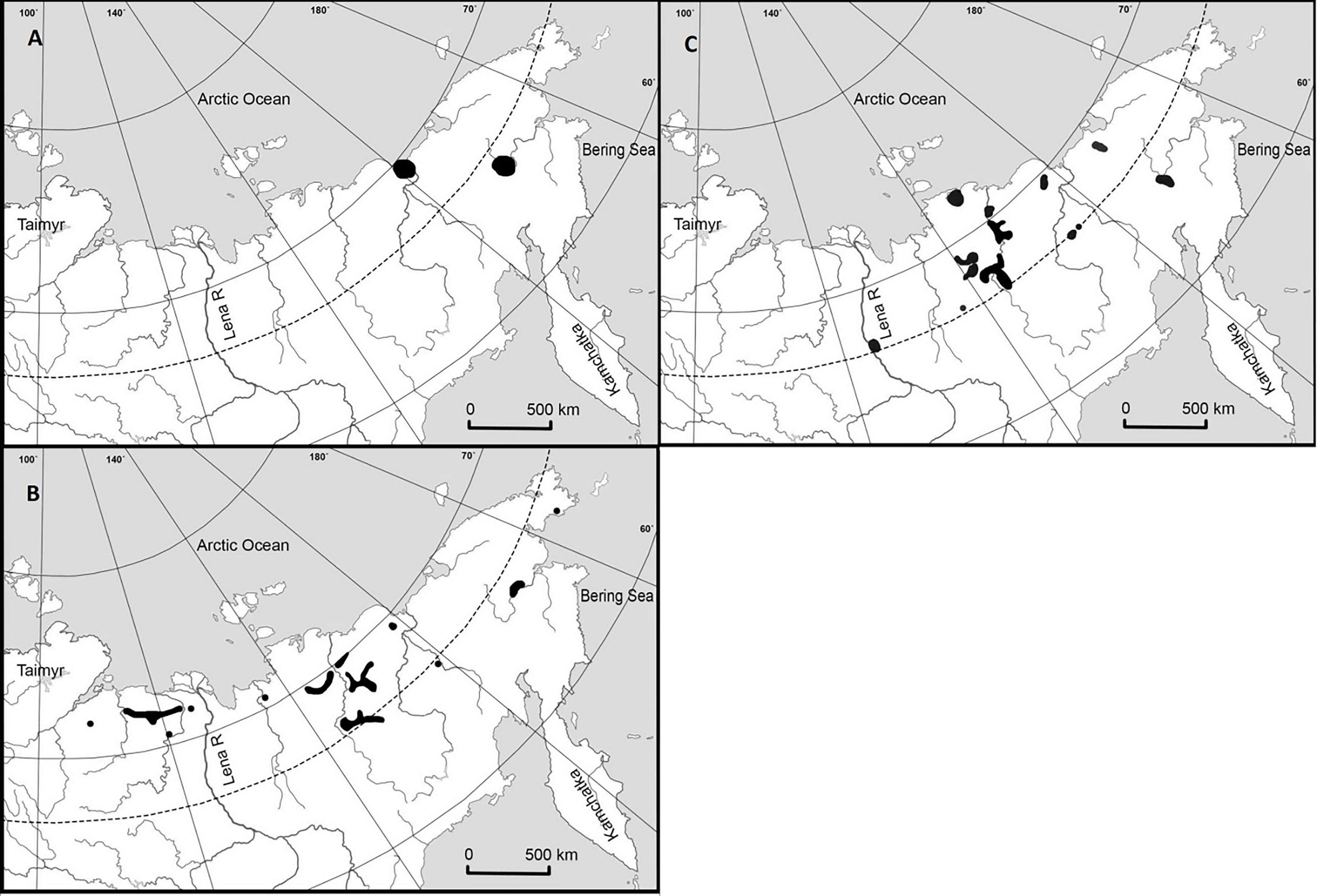

Supplement: Supplementary file 2 — Figure S1 [file ECE3-11-4126-s002.png]
